# Analog synthetic biology

**DOI:** 10.1098/rsta.2013.0110

**Published:** 2014-03-28

**Authors:** R. Sarpeshkar

**Affiliations:** Analog Circuits and Biological Systems, Research Lab of Electronics, Massachusetts Institute of Technology, Cambridge, MA 02139, USA

**Keywords:** analog computation, synthetic biology, cytomorphic, logarithmic computation, probabilistic computation, bioenergetics

## Abstract

We analyse the pros and cons of analog versus digital computation in living cells. Our analysis is based on fundamental laws of noise in gene and protein expression, which set limits on the energy, time, space, molecular count and part-count resources needed to compute at a given level of precision. We conclude that analog computation is significantly more efficient in its use of resources than deterministic digital computation even at relatively high levels of precision in the cell. Based on this analysis, we conclude that synthetic biology must use analog, collective analog, probabilistic and hybrid analog–digital computational approaches; otherwise, even relatively simple synthetic computations in cells such as addition will exceed energy and molecular-count budgets. We present schematics for efficiently representing analog DNA–protein computation in cells. Analog electronic flow in subthreshold transistors and analog molecular flux in chemical reactions obey Boltzmann exponential laws of thermodynamics and are described by astoundingly similar logarithmic electrochemical potentials. Therefore, cytomorphic circuits can help to map circuit designs between electronic and biochemical domains. We review recent work that uses positive-feedback linearization circuits to architect wide-dynamic-range logarithmic analog computation in *Escherichia coli* using three transcription factors, nearly two orders of magnitude more efficient in parts than prior digital implementations.

## 1. Introduction, motivations and overview

Every living cell within us is a hybrid analog–digital supercomputer that implements highly computationally intensive nonlinear, stochastic, differential equations with 30 000 gene–protein state variables that interact via complex feedback loops. The average 10 μm human cell performs these amazing computations with 0.34 nm self-aligned nanoscale DNA–protein devices, with 20 kT per molecular operation (1 ATP molecule hydrolysed), approximately 0.8 pW of power consumption (10 M ATP s^−1^) and with noisy, unreliable devices that collectively interact to perform reliable hybrid analog–digital computation [1]. Based on a single amino acid among thousands of proteins, immune cells must collectively decide whether a given molecule or molecular fragment is from a friend or foe, and if they err in their decision by even a tiny amount, autoimmune disease, infectious disease, or cancer could originate with high probability every day [2]. Even at the end of Moore's law, we will not match such performance by even a few orders of magnitude [1,3].

The field of synthetic biology attempts to transfer engineering design principles and experimental techniques into rational biological design [4–8]. It represents the ultimate limit of Moore's law: computation with the molecules themselves at the nanoscale through the use of controlled biochemistry and biophysics. Such an approach can blend ‘fabrication’ and ‘computation’ in a seamless fashion. The self-organizing amorphous soup in a cell processes information while it destroys, repairs and rebuilds the structures needed to do so. It is remarkable that it does so through a self-aligned nanotechnology with no explicit wiring. Instead, chemical binding among specific molecules serves to ‘implicitly wire’ them together and causes them to interact via chemical reactions. These reactions cause transformations of state, which are necessary for computation to occur.

While significant progress has been made w.r.t. fundamentals and applications in the field of synthetic biology, it has failed to scale significantly in complexity over more than a decade [9,10]. One important reason for this failure has been its overemphasis on digital paradigms of thought: because digital design is relatively straightforward and scalable, and because molecules and atoms are discrete, it is logical to assume that engineering biology as we engineer switches and logic gates today will bear fruit. The sheer force and powerful success of digital computation over the past few decades has been impressive. Nevertheless, we must not forget that digital computation has not offered an effective paradigm for computing efficiently and precisely with noisy and unreliable devices; that multi-logic-gate computations can impose significant metabolic or toxicity burdens on cells owing to their need to use a lot of parts and power; that the fact that there are five to six orders of magnitude fewer genes per cell than digital transistors per chip means that using genes to only perform logic is likely not an efficient way to attain high complexity; that a library of ‘digital parts’ with good on–off ratios, low standby power consumption and low crosstalk does not exist in biology; that the computing basis functions in cells are not really logic functions and abstracting them as such compromises computational efficiency; and that logic basis functions are not the only universal computation primitives.

Nature is not purely digital. While molecules are discrete and digital, all molecular interactions that lead to computation, e.g. association, transformation and dissociation chemical reactions, have a probabilistic analog nature to them. Depending on one's point of view, computation in a cell is owing to lots of probabilistic digital events or owing to continuous analog computation with noise. Both views are equivalent. Indeed, the noise in analog systems is related to the Poisson rate of the underlying probabilistic digital events; the shot noise of thermally generated diffusion currents caused by these Poisson processes generates noise in all analog systems [1,11].

Synthetic biology has now recognized that the signals in cells are stochastic (noisy) and analog (graded) in their nature [12]. The ‘1's and ‘0's of today's digital computers are useful abstractions of the analog signals in cells, but are often an oversimplification. Furthermore, as in analog circuits, the wiring of the output of one circuit to the input of another leads to ‘loading’ interactions that degrade overall function and prevents simple modular digital abstractions from being effective [13]. While logic basis functions and positive-feedback loops are certainly used by cells to make irreversible decisions, to organize sequential computation and to perform signal restoration, analog computation is extremely important for the cell's incredible efficiency w.r.t. the use of energy, time and space [1,14]. The efficiency arises because analog computation can use powerful input–output basis functions in the technology for addition, multiplication, exponentials, logarithms, power laws and spatio-temporal filtering, which are already naturally present in the differential equations of physics and chemistry. Therefore, it does not need to re-invent these input–output basis functions from scratch with logic. For example, the production fluxes of two genes that encode the synthesis of a common output protein automatically perform addition via a molecular version of Kirchoff's current law without the need for tedious logic; the binding of two molecules provides a basis function for multiplication; the binding of identical molecules in a dimer provides basis functions for computing squares or square roots; molecular degradation naturally provides basis functions for temporal filtering; diffusion naturally provides a basis function for spatial filtering.

The pioneers of digital computation, John von Neumann and Alan Turing, appreciated the great power of analog computation and were investigating it intensely to cope with the limitations of using only logic to compute. Near the end of their lives, they were working on understanding analog computation in brains [15] and in cells [16], respectively. Analog computation has long been appreciated to be important in the brain [17], but its importance in cells has been greatly underappreciated. The power of the analog wave function in quantum mechanics enables quantum computers to solve problems that no digital computer can [18].

In this article, we begin by describing analog circuit schematics for a single gene, which provide a foundation for the rest of the article and a quick introduction to the computational basics of cell biology. Then, we review the pros and cons of analog versus digital computation with a focus on computation in living cells. In particular, we extend a prior analysis [1,14] to a simple exemplary computation in *Saccharomyces cerevisae* (yeast) cells, namely that of adding two numbers in an analog versus a digital fashion. This simple example will help us to analyse general trade-offs w.r.t. energy consumption or w.r.t. the number of molecules needed to implement addition at a given level of computational precision. The analysis will show that, below a certain crossover computational precision, it is highly advantageous to compute in an analog fashion to reduce the energy, part count or number of molecules (and thus volume or space) needed for the computation. It is therefore not surprising that cells exploit analog computation to perform their moderate-precision computations. Other work has also shown that *collective analog* computation can use several moderate-precision interacting analog units to architect computations of arbitrary precision, e.g. 16-bit addition via four interacting 4-bit-precise analog computational units [1,19], on an experimental silicon chip [20]. Collective analog systems can also architect arbitrary complex computations through interactions of moderate-precision analog computational units in the brain and in the cell. Indeed, cellular and neural computations bear 13 similarities to each other from a hybrid analog–digital point of view [1].

In analog computation, every signal and every device are not reliable, but important final or decisive outputs are. To attenuate noise, analog computation invariably relies on feedback loops, the wise use of energy, time or space resources to improve precision via averaging at critical state variables and reference inputs, learning and adaptation, nonlinearities, such as thresholding or digitization, or invariants and attractors in the analog dynamical system that cause it to equilibrate to restorative discrete outputs. Though noise limits information capacity, it can sometimes be beneficial for searching intractable combinatorial spaces and for improving the detection of signals by phenomena such as stochastic resonance.

How do we transfer analog circuits in electronics to analog circuits in cells? Fortunately, there is a deep connection between electronics and chemistry, which greatly aids the design of analog circuit motifs and analog computation in synthetic biology. This deep connection arises because there are astounding similarities between the equations that describe noisy electronic flow in subthreshold transistors and the equations that describe noisy molecular flow in chemical reactions, both of which obey the laws of exponential thermodynamics [1,21]. Therefore, circuit motifs from the electronic domain are useful for creating circuit motifs in biology and vice versa in a *cytomorphic* fashion, as figure 3 shows [1]. A recent paper [22] showed that log-domain analog circuits in living *Escherichia coli* bacterial cells, which were inspired by log-domain electronic circuits, could efficiently compute logarithms, add, subtract, divide and do square-root-like computations with less than three genetic parts. By contrast, a prior *in vitro* 2-bit-precise digital square-root computation required 130 DNA-based parts [23]. Another circuit described in [22] was able to accurately compute the logarithmic ratio of two molecular concentrations over four orders of magnitude. The latter circuit used novel positive-feedback linearization circuits, similar to those used in my laboratory in log-domain subthreshold electronic amplifiers in the past [24]. The genetic circuits described in [22] may have wide applications for wide-dynamic-range molecular sensing, complex computation with few parts in biotechnology and medicine, and for the fine control of gene expression.

The analog circuits approach described in this article may enable large-scale design and analysis in synthetic and systems biology, which is faithful to how messy analog biology works, quite different from clean, well-defined digital design. It may enable several applications in synthetic biology, wherein, just as in electronics today, all applications benefit from low-part-count, low energy usage and clever analog feedback loops to improve performance. For example, synthetic biological circuits will be increasingly important in finding cures for diabetes, cancer, autoimmune, infectious and other diseases [25] wherein sophisticated synthetic feedback loops will have to compensate for error in natural feedback loops; synthetic molecular circuits are also important in biomolecule and pharmaceutical manufacturing [26] wherein efficient resource consumption and economic viability are closely tied; in energy production and environmental remediation [27,28] where efficient bacterial operation and fine control of gene expression is beneficial; in artificial fuel generation [29] where energy efficiency is paramount; in environmental pathogen or pollutant sensing where robust single-molecule analog amplification and wide-dynamic-range sensing is beneficial; in the design of ‘nanorobots’ and biomechanical devices [30] where analog feedback sensing and actuation loops could enable synthetic target-tracking behaviour. Analog synthetic biology can serve to make synthetic computation practical because it does not impose man-made views of what computation should be in the cell, but computes in a fashion that is similar to the way the cell itself computes.

Section 2 provides an analog circuit schematic of a gene that is useful for accurately representing steady-state, dynamic and stochastic phenomena. The success of analog circuit design arises in large part from having efficient pictorial representations that are more intuitive to humans than reams of differential equations. Intuition aids design. The analog circuit schematics nevertheless preserve most of the needed mathematical information in the equation as labelled parameters, such that analysis can aid design. Section 3 discusses the pros and cons of analog versus digital computation, first intuitively, then quantitatively. Section 4 discusses the deep similarities between electronics and chemistry, which can enable powerful cytomorphic circuit design in cells owing to the presence of a common logarithmic electrochemical potential. Section 5 provides examples of such circuit design using log-domain genetic circuits. Section 6 concludes with a summary of seven benefits of analog computation in synthetic biology.

## 2. Analog circuit schematic of a genetic promoter

**Figure 1.**
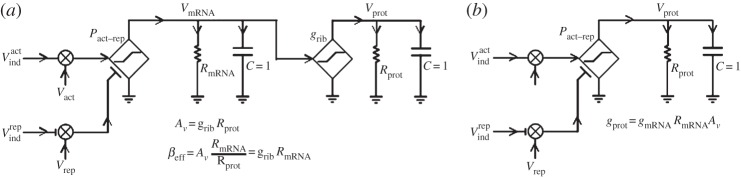
Analog schematic for genetic circuits. (*a*) An electrical equivalent circuit accurately represents the noise, dynamics and stochastics of DNA–protein circuits. (*b*) A simplification of the circuit in (*a*) that neglects mRNA dynamics.

Figure 1*a* shows an analog circuit schematic of a genetic circuit with two inputs: one activator, *V*_act_, which enhances the rate of gene transcription (expression) and one repressor, *V*_rep_, which suppresses the rate of gene transcription. The gene transcription initiates from a ‘promoter region’ on the DNA, *P*_act−rep_, near which these molecules bind and interact with the DNA reading machinery of the cell. The diamond-shaped dependent current generator, *P*_act−rep_, outputs an *mRNA* transcription current that is controlled by these ‘transcription factor’ inputs via an ‘analogic’ basis function with both linear analog and saturating digital regions of operation (the minimum and maximum expressions) [21]. The mRNA transcripts accumulate on the capacitor *C* (which always has *C*=1 [1]) to create *V*_mRNA_, the molecular concentration of mRNA.The resistor *R*_mRNA_ degrades the mRNA such that the synthesis current from the DNA promoter *P*_act−rep_ eventually balances the degradation current and *V*_mRNA_ equilibrates at a steady-state value. The transcription factor *V*_act_ is itself activated by an inducer input, *V* ^act^_ind_, which binds to it via the ‘multiplier’ symbol, and activates it to bind DNA and stimulate transcription. Similarly, the transcription factor, *V*_rep_, which normally binds DNA and represses transcription is de-repressed by *V* ^rep^_ind_, such that the net effect of *V* ^rep^_ind_ is to also stimulate gene expression in this example. Repression is always indicated by the T-shaped symbols and activation is always indicated by arrow inputs at the border of the multiplier or at the border of the dependent current generator. Inducer inputs are typically small-molecule external inputs to the cell that diffuse into it and bind internal transcription factors within the cell. In bacteria and yeast, it is not common to have more than two controlling inputs at a DNA promoter, but mammalian cells can have several controlling inputs. The dependent generator will have as many inputs as the DNA promoter.

The dependent current generator on the right end of figure 1*a* ‘translates’ *V*_mRNA_ via ribosomal machinery in the cell to create a synthesis protein current, which accumulates on a capacitor to create *V*_prot_, the molecular concentration. The resistor *R*_prot_ degrades the protein such that the synthesis translation current balances the protein degradation current and *V*_prot_ reaches a steady-state value. The equations in figure 1*a* define *A*_*v*_ and *β*_eff_, which determine the gain from *V*_mRNA_ to *V*_prot_ and a noise factor similar to the current gain of a bipolar transistor, respectively [1]. We discuss the importance of *β*_eff_ for determining noise in §3. The dynamics and stochastics (noise) of the circuit of figure 1*a* faithfully capture experimental dynamic data [31] as well as noise data [32] provided that the shot noise of the molecular-dependent generators and resistors is accounted for correctly [1]: the resistor noise, unlike that in a normal resistor [11], is simply the shot noise of the net current flowing through it [1]. The current gain *β*_eff_ in figure 1*a* corresponds to a ‘burst factor’ that has been experimentally measured [32]. Compact 8-transistor analog circuits, which are current-mode circuit versions of figure 1*a*, create analogic basis functions that are mathematically exact representations of prior models [21]. They also match experimental input–output data gathered from non-pathogenic *E. coli* [21].

As *R*_prot_ is typically much greater than *R*_mRNA_, it is common to assume that the mRNA dynamics are relatively instantaneous compared with protein dynamics [31]. Thus, the approximate circuit schematic of figure 1*b*, which is a simplified representation of figure 1*a* without m*RNA* dynamics and without explicit m*RNA* state variables, is significantly more convenient and usually accurate. It allows one to focus on a protein-input–protein-output point of view. In this article, our noise analysis in §3 is based on the accurate figure 1*a*; all other genetic circuit schematics are drawn similar to that of figure 1*b*.

## 3. Analog versus digital

We begin with an intuitive comparison of the pros and cons of analog versus digital computation presented in table 1.

**Table 1.** Intuitive analog versus digital comparison.

	analog	digital
	(1) compute on a continuous set, e.g. [0,1], graded protein production from low to a maximum level	(1) compute on a discrete set, e.g. {0,1}, protein produced at a maximum level or not present at all
	(2) the basis functions for computation arise from the physics and chemistry of the computing devices such that the amount of computation squeezed out of a single genetic, RNA or protein circuit is high	(2) the basis functions for computation arise from the mathematics of Boolean logic such that the amount of computation squeezed out of a single genetic, RNA or protein circuit is low
	(3) one wire, channel or state variable represents many bits of information	(3) one wire, channel or state variable represents one bit of information
	(4) computation is sensitive to the parameters of the molecular circuits	(4) computation is less sensitive to the parameters of the molecular circuits
	(5) noise owing to thermal fluctuations in molecular devices	(5) noise owing to round off error and temporal aliasing
	(6) signal is not restored at each stage of the computation	(6) signal is restored at each stage of the computation
	(7) robust at final and decisive outputs	(7) robust in every device and signal

Table 1 presents that analog computation emphasizes efficiency while digital computation emphasizes robustness. Robustness–efficiency trade-offs are ubiquitous in all engineering systems [33] and are at the heart of all good engineering design. A mathematical understanding of noise is essential for quantifying the intuition behind table 1. Therefore, we shall begin by deriving some important results regarding molecular noise in cells.

### (a) Molecular noise in cells

There are a lot of experimental measurements and numbers available for *S. cerevisae*. Hence, we shall use it as our representative cell. For any gene, the Poisson shot noise of molecular flux that produces mRNA from genetic DNA and of molecular flux that produces protein from the mRNA is averaged over the bandwidth of an RC-like protein degradation filter to generate a mean protein copy number *N*_prot_ [1,31]. It also generates fluctuations around this mean with a mean square value of 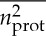 [1,32]. In figure 1*a*, *N*_prot_ and 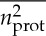 correspond to the mean value and fluctuations of *V*_prot_, respectively.

Typically, mean mRNA copy numbers, *N*_mRNA_, are low (0.1 to 130 in *S. cerevisae*) [34–37], while protein copy numbers are high (50–10^6^ in *S. cerevisae*), and mRNA degradation time constants, *τ*_mRNA_, are small (3–90 min in *S. cerevisae*) [35] while protein degradation time constants, *τ*_prot_, are large (10–1000 min in *S. cerevisae*) [38]. Therefore, there is molecular gain from mRNA to protein, *A*_*v*_=*N*_prot_/*N*_mRNA_ or *V*_prot_/*V*_mRNA_ in figure 1*a*, which amplifies the mRNA noise content in the protein signal. However, owing to the averaging caused by an effective bandwidth reduction factor of *τ*_mRNA_/*τ*_prot_, there is also a reduction in mRNA noise content in the protein signal in figure 1*a*. The total noise of the protein signal is owing to the net mRNA noise content as well as that owing to the intrinsic shot noise of the Poisson protein molecular flux. That is,
3.1
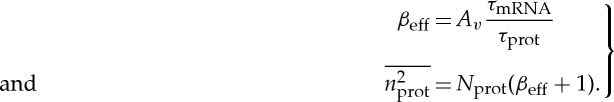
The equations in figure 1*a* show circuit parameters for *β*_eff_ and *A*_v_. These circuit parameters reproduce equation (3.1) exactly with *τ*_mRNA_=*R*_mRNA_*C*, *τ*_prot_=*R*_prot_*C* and *V*_prot_=*N*_prot_. The output signal-to-noise ratio *S*_N_ is then given by
3.2
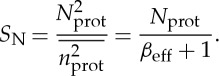
The equivalent informational bit precision [39] is given by
3.3
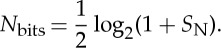
Equations (3.2) and (3.3) reveal that, to encode an output signal at high precision (large *S*_N_ or large *N*_bits_), the molecular protein copy number *N*_prot_ and consequently the mRNA copy number need to increase in proportion with *S*_N_:
3.4
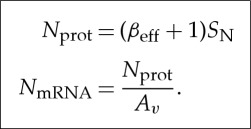


### (b) Signal power consumption

Protein and mRNA molecules have to be constantly synthesized to counter their degradation, and this synthesis consumes power. The synthesis power is provided by the hydrolysis of several molecules of adenosine triphosphate (ATP), the universal energy-providing molecular currency of cells. ATP hydrolysis enables various processes in transcription and translation to move forward in a nearly irreversible fashion [2]. The hydrolysis of one molecule of ATP provides nearly 20 kT [40]. The energy cost of synthesizing an mRNA molecule in ATP is *E*_mRNA_≈30*L*_mRNA_ [37], where *L*_mRNA_ is the length of the mRNA molecule in nucleotides, with a median value of *L*_mRNA_=1474 nucleotides for yeast [34,36]. Similarly, the energy cost of synthesizing a protein molecule in ATP is *E*_prot_≈50*L*_prot_ [37], where *L*_prot_ is the length of the protein molecule in amino acids, with a median value of *L*_prot_=385 amino acids in yeast. Hence, the power consumption needed to charge or maintain a protein signal level of *N*_prot_ is given by
3.5
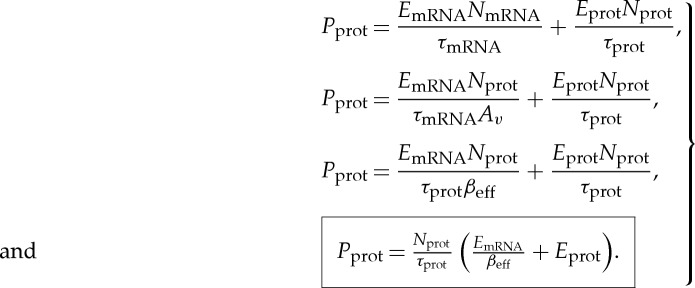


### (c) Minimization of power consumption

If we substitute for *N*_prot_ in the boxed expression in equation (3.5) with its value in equation (3.4), we find another important relationship
3.6
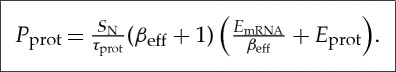
Equation (3.6) is a classic example of a *resource–precision* equation [1,14] that quantifies how power consumption (the resource) increases as the speed (1/*τ*_prot_) and the precision (*S*_N_) of signals in a computation increase. Such resource–precision relationships are universal and can be found in neurobiological, electrical, mechanical and all systems [1]. Intuitively, to be fast and precise, a scenario that maximizes information, a system consumes power. Hence, universal information-based principles to reduce power consumption in any system from brains to electronic systems architect methods to reduce speed or precision at local state variables while maintaining system speed and precision [1,41]. For example, slow-and-parallel systems with moderate local precision lead to very power efficient systems, both in the brain and in electronics [1]. Equation (3.4) is another example of a resource–precision equation but the resource here is molecular copy number rather than power consumption. In electronic systems, resource–precision equations relate power consumption and area consumption on chips to speed and precision [1,14].

Equation (3.6) suggests that, for a given precision (*S*_N_) and speed (1/*τ*_prot_), there is an optimum value of *β*_eff_ that reduces power consumption. Simple differentiation reveals that this optimum occurs when
3.7
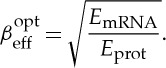
At this optimum, we can substitute for *β*_eff_ in equation (3.5) or in equation (3.6) to determine the power consumption
3.8
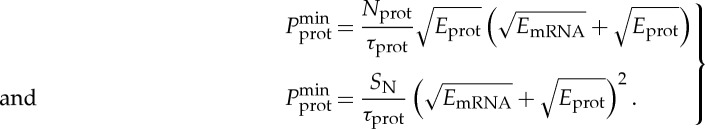
From equation (3.1), for a given speed of operation, 1/*τ*_prot_, the optimum *β*_eff_ can be attained either by varying the gain *A*_*v*_ if the time constants are fixed, or by varying *τ*_mRNA_ if *A*_*v*_ is fixed. If *τ*_mRNA_ is fixed, and 1/*τ*_prot_ is increased to improve the speed of the computation, then *A*_*v*_ must be lowered to maintain *β*_eff_ at its optimal value.

### (d) An analog molecular adder

To implement a molecular adder via analog computation is simple: we have the two inputs to be added; each regulates the expression of a common output protein from independent genetic promoters. During synthesis, the two molecular fluxes will automatically add to create the common output protein, which is the answer. To enable linear analog operation and avoid saturated digital operation, the input proteins must operate at a concentration that is well below *K*_d_, the binding constant of the DNA promoter. A large *K*_d_ can be architected by using weak, mutated or multiple-binding-site promoters [22,31]. As the input and output concentrations increase, the input and output noise reduce, the precision of the computation improves, but at the price of higher molecular count and higher power consumption. The computation only requires two genetic parts, independent of precision or molecular count, which makes it practical to implement. To estimate the costs of this computation, we shall assume, for simplicity, that the input and output molecular counts are equal (promoters with a net gain of 1), which enable both the precision of the input and output to concomitantly scale with the overall needs of the computational precision. A proportionate gain scaling between the input and output is also possible but adds complexity while obscuring the key insights that we want to emphasize.

Analogous to how mRNA noise flux propagates to protein outputs and increases noise, the input noise flux propagates to the adder output. Thus, the adder will have twice the squared noise as would be expected from its output molecular count alone. Therefore, to preserve the *S*_N_ all molecular counts must be increased by a factor of 2 at the input and output, leading to a four times larger total molecular count for the same *S*_N_. Thus, the fundamental resource–precision equations for one signal, equations (3.4) and (3.5), cause the resource–precision equations for the molecular count, *N*_A_, and power consumption, *P*_A_, of the analog adder to be
3.9
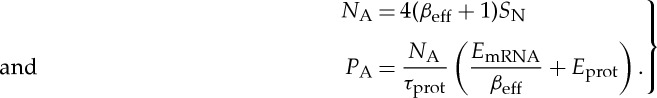
Circuit noise-analysis techniques described in [1] can be used to derive resource–precision equations similar to equation (3.9) for arbitrarily complex analog computations.

### (e) A digital molecular adder

An *N*-bit digital molecular adder requires 1 half-adder logic circuit (binary addition without carry for the least significant bit), *N*−1 full-adder circuits (binary addition with carry for the *N*−1 other bits) and two *N*-bit inputs. Appendix A presents that the total number of logic gates, each of whose outputs requires energy-consuming molecular synthesis, as well as 2*N* inputs, adds up to 17 *N*−7 net protein (and associated mRNA) state variables for *N*≥2. If *N*=1, six protein state variables are needed.

At high ‘1’ levels, protein synthesis occurs at a high rate, and at low ‘0’ levels, protein synthesis occurs at a lower rate. To minimize the power consumption, digital logic gates must have as small a protein copy number for high values as possible. At low values, the ‘leakage’ or ‘basal transcription’ in the genetic circuits will then limit practical implementations to 100-fold or 1000-fold lower protein copy numbers (*N*_mRNA_ varies from 0.1 to 130 in yeast and protein copy numbers then vary proportionately [34–37]). However, the ‘1’ value must be sufficiently large such that it enables saturated operation, which is necessary for robust digital signal restoration in signal cascades, for a low bit error rate owing to sufficient noise margins between ‘1’ and ‘0’ values, for enabling robust fan-out that is not compromised by loading, and for robust operation in spite of *K*_d_ variations among gates. The parameter *K*_d_ is an equilibrium chemical binding constant that effectively behaves as a threshold voltage in the digital operation.

A reasonable design choice is to use a ‘1’ value that is at least 10 times the *K*_d_ of the logic promoter such that multiple logic gates can function together in fan-out or cascade configurations with good noise margins and in spite of *K*_d_ variations among gates. Note that *K*_d_ variations among gates can be static or dynamic owing to loading and crosstalk. As in all engineering systems, a robustness–efficiency trade-off exists: a large ‘1’ value will robustly guarantee saturation and signal restoration, good noise margins with low error rate, robust fan-out and robustness to *K*_d_ variations but will require higher power consumption to encode the ‘1’.

Measured *K*_d_'s for signalling proteins are usually in the 30 nM–10 μM range [42], and some sources report about 10 nM [43]. We shall assume that 10 nM is practical for all of our logic gates, and that all operate at a ‘1’ value that is 10 *K*_d_ and at a ‘0’ value that is at least 200 times lower. As yeast is approximately 200 μm^3^ in volume [31,43], 200 molecules correspond to 1 nM and 2000 molecules correspond to 10 nM (near the median copy number of most proteins in yeast [34–37]). Thus, a ‘1’ value corresponds to 20 000 molecules. If *N*_digHI_=20 000, then *N*_digLO_=20 000/200=100, which is about two times the lowest measured copy numbers in yeast [34–37]. The exact value of *N*_digLO_ does not matter too much as long as it is sufficiently low.

Many signalling proteins require at least dimerization to bind, which doubles molecular count and power consumption for the same *K*_d_; but, we could reduce the ‘1’ value to operate at 5*K*_d_ owing to higher digital slopes, thus halving molecular count and power consumption. Thus, we shall, for simplicity, ignore dimerization and polymerization effects that are, to first order, neutral w.r.t. resource consumption in our analysis. For simplicity, we shall also assume that any logic gate operates at saturated values that are the same as that of its inputs such that logic gates can be easily composed and cascaded similar to that in today's electronic systems. Otherwise, digital implementations will require molecular gain and attenuation between logic stages. So, our assumption is generous to digital implementations.

Appendix A presents that the delay of the digital adder is approximately (*N*+2)*τ*_prot_. Therefore, for the same speed as a parallel analog adder, each logic stage of the digital adder must decrease its degradation time constant, *τ*_digN_, according to
3.10
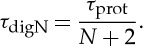
From equation (3.1), to operate at the optimal *β*_eff_ given by equation (3.7), the gain *A*_*v*_ can be adjusted to *A*_*v*digN_ according to
3.11
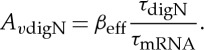
If the probabilities of low and high values in the adder are *p*_low_ and *p*_high_, respectively, with corresponding values of *N*_digHI_ and *N*_digLO_ for the protein state variables, then the resource–precision equations for the molecular count, *N*_D_, and the power consumption, *P*_*D*_, of the digital adder can be computed to be
3.12
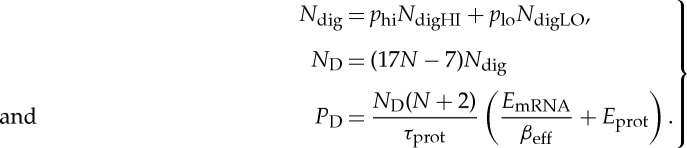
For most computations, *p*_hi_=*p*_lo_=0.5 if we average across all possible inputs. Random additive inputs to our adder will generate equal probabilities of ones and zeros.

### (f) Analog addition versus digital addition

The resource–precision equations for analog addition are described by equation (3.9), while those for digital addition are described by equation (3.12). To compare the costs of analog versus digital addition at the same speed (1/*τ*_prot_) and precision (*S*_N_ for analog and *N* for digital), we use equation (3.3) to convert *S*_N_ to an equivalent number of bits *N*_bits_. With the analog and digital precision now in the same units (*N* for digital and *N*_bits_ for analog), we can compare analog and digital computation w.r.t. their resource consumption of molecules or power consumption. Table 2 lists a set of parameters for yeast used to make this comparison [34–38,44], which were chosen to minimize power consumption in the analog and digital domains.

**Table 2.** Parameters used for molecular addition.

parameter	value
*τ*_mRNA_	3 min
*τ*_prot_	1000 min
*N*_digHI_	20 000
*N*_digLO_	100
*p*_hi_	0.5
*p*_lo_	0.5
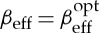	2.49

**Figure 2.**
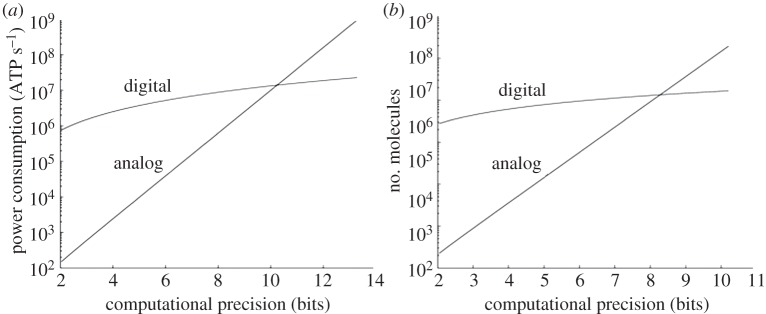
Analog versus digital computation in cells. (*a*) The figure shows the power costs for doing addition in cells with a genetic circuit. (*b*) The figure shows the molecular protein number required for doing addition in cells with a genetic circuit.

Figure 2*a* reveals that the costs of analog power consumption in ATP s^−1^ are significantly less than those of digital power consumption even at nearly 10 bits of precision. At this crossover precision, the power consumption is a staggering 10 million ATP s^−1^, so high that a yeast cell will be using almost its entire power budget to add two numbers at 10-bit precision! It is not surprising then that cells do not waste their energy doing highly accurate digital computation as computers do. The figure also implies that a synthetic biological circuit that just has 163 simple two-input deterministically robust logic gates is not practical. Thus, deterministic digital approaches to synthetic computation have barely scaled over a decade [9] and seem unlikely to in the future. Digital computation will likely have to be probabilistic and therefore behave more as noisy analog computation with a lower value of *N*_digHI_. A lower value of *N*_digHI_ will necessarily make digital computation less robust and more probabilistic as we discuss in §3*g*. Probabilistic ‘dithering’ converts digital black-and-white pixels in an image to greyscale pixels. Thus probabilistic digital operation will have similarities to noisy analog operation, which is in fact noisy precisely because many probabilistic digital Poisson events constitute its value [1,11].

Figure 2*b* shows that the costs of analog addition in total protein molecular number are also significantly less than those of digital addition even at nearly 8 bits of precision. At this crossover precision, a staggering 1 million protein molecules are involved in addition. This number is about 1 in every 1000 protein molecules in yeast [31], which may be possible in the analog approach and harder in the digital approach (because 129 logic gates are needed). However, from [44], this 1/1000 change is about three orders of magnitude higher than the change needed to impact evolution in a yeast population. Therefore, the molecular-count costs of adding at 8-bit precision in a cell are still very expensive. At the 8-bit crossover in figure 2*b*, figure 2*a* shows that the corresponding analog power consumption is almost an order of magnitude less than digital power consumption.

High molecular copy numbers and high power consumption are characteristic of high-precision computation. Both lead to toxicity in the cell: high protein copy numbers can cause inadvertent binding of high-copy-number molecules to molecules in essential pathways and interfere with their function via competitive binding. High power consumption generates reactive oxygen species that lead to oxidative stress, cellular damage and cell death. So, high power consumption is not just an undesirable feature in cells as it is in some electronic systems: it actually leads to death. In some electronic systems, especially digital ones, high power consumption can lead to catastrophic failure of portions of a chip via thermal runaway effects.

### (g) Analog versus digital

Do the lessons learned from the example of addition generalize to other computations? Are these lessons sensitive to our parameter values and assumptions? All computations have analog–digital crossover curves similar to those in figure 2*a*,*b* but the exact crossover precision depends on the computational task. Tasks that can be performed with fewer analog parts push the crossover point to higher precision [1,14]. Feedback loops in analog computation that correct for error push the crossover to higher precision [1]. Parameter changes can certainly shift the location of the crossover point and are different in different technologies—in electronics, in neurobiology and in cells [1,14].

As an example of how parameters can shift the analog–digital crossover point, let us assume that *N*_digHI_=2 K instead of 20 K such that digital power consumption is reduced by nearly 10 times. The analog–digital crossover point for power consumption will then be near 7 bits rather than 10 bits for addition, which is still very precise for a cell. In practice, there may be several problems associated with this lower value of *N*_digHI_: with *N*_digHI_=2 K, we require operation at *K*_d_=1 nM for all gates in the digital adder. The low *K*_d_ requires significant DNA–protein engineering to improve chemical binding for all adder molecules, and also leads to a significant slowing of transcription-factor binding to DNA analogous to similar trade-offs seen in translation [45]. Higher noise caused by a lower *N*_digHI_ increases the bit error rate in each channel, making the overall digital computation more probabilistic. As the median protein copy number in yeast is nearly 2000 [34–37], the lower *N*_digHI_ may also lead to significant crosstalk owing to competitive binding with background molecules. As *β*_eff_ is not likely at its optimal value for all digital signal variables as we have assumed, each of them will be noisier, such that the lower *N*_digHI_ may significantly increase bit error rate. Finally, even with *N*_digHI_ at a 10 times lower value, nearly 10% of the cell's power budget would just be spent on 7-bit addition, compromising its other functions severely. At a 100 times lower value of *N*_digHI_=200, digital operation will be highly probabilistic owing to insufficient noise margins and it would require significant engineering to reduce *N*_digLO_ to values near 20 to achieve on–off ratios of just 10: protein copy numbers below 50 are extremely rare in yeast [34–37]. So our choice of *N*_digHI_=20 K to get yeast cells to perform digital addition with 163 gates in a deterministic and robust digital fashion appears to be reasonable. By contrast, as the analog approach only requires two genes, it is significantly easier to engineer and optimize.

The fundamental reason for the analog–digital crossover does not lie in issues having to do with parameters and numbers. It really lies in information theory: information is coded across many 1-bit-precise interacting computational channels in the digital approach but on 1 multi-bit computational channel in the analog approach [14]. At low informational precision, logic basis functions simply cannot compete with the richer basis functions of analog computation that can process all the bits at once in parallel and just automatically solve the task, e.g. by using Kirchoff's current law for addition or chemical binding for multiplication. At high precision, however, the fundamental laws of large numbers and thermodynamics require the noise per computational channel to be too low in analog computation: the low-noise requirement drastically increases the costs of energy (more energy enables more molecules to be produced), time (more time allows for more noise reduction via averaging) or space (molecular count or parallel and redundant architectures cost volume) for analog computation. Digital computation wisely distributes the information and information processing over many channels that interact with each other to preserve information, e.g. in the case of addition via a carry. No one channel is required to perform heroically in digital computation. Thus, the polynomial scaling of resource costs with *N*=*N*_bits_ in digital computation, e.g. in equation (3.12) or equivalently its logarithmic scaling with *S*_N_ in equation (3.3), eventually always outcompete analog computation's polynomial scaling with *S*_N_, e.g. in equation (3.9).

From analyses such as this one and others, we have concluded that the most efficient and scalable form of computation for arbitrary precision and complexity is a hybrid of analog and digital computation termed as *collective analog* computation [1,14,19,20]. In this form, several moderate-precision analog computational units interact to preserve and process information as many interacting analog neurons in the brain do. Certainly, one often uses digital signals for decision-making, signal restoration and communication within and among the analog units. For example, the first 16-bit (and scalably) precise analog adder in electronics works in this fashion on an experimental silicon chip [20]. Given current experimental data, it is likely that cells use many interacting 2- to 5-bit-precise analog computations to compute. Neurons also use distributed computation with moderate-precision analog components to reduce their significant energy costs [14,46,47].

Hybrid analog–digital architectures termed as *hybrid-state machines* (HSMs) [1] are useful for describing computations in cells in development or cell cycle pathways [2]. These machines provide a methodology for gene–protein networks to compute in a sequential analog fashion and generalize the notion of finite state machines to the hybrid analog–digital domain. HSMs also provide a good framework for describing spiking neuronal computation [19,20]. Indeed, there are 13 similarities between gene–protein and neural computation from a hybrid analog–digital point of view [1], and *in vitro* molecular computation has attempted to engineer neural-network-like computations [48].

## 4. Deep connections between electronics and chemistry

**Figure 3.**
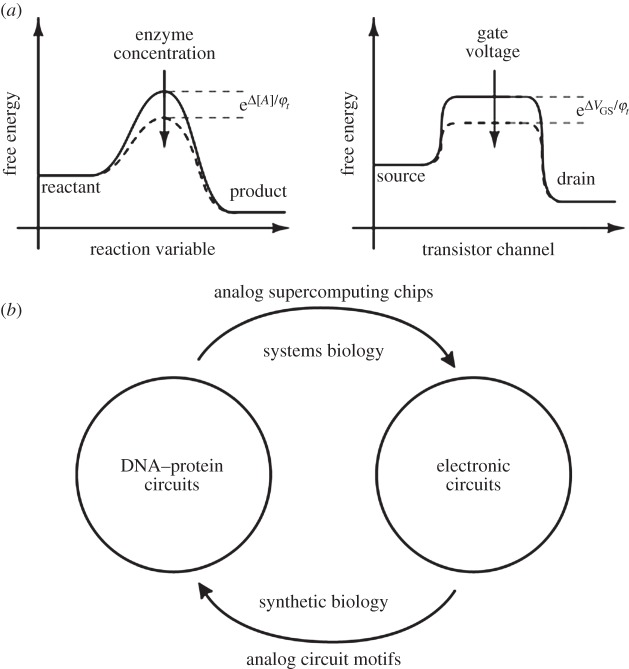
The cytomorphic mapping. (*a*) The figure shows the deep connections between electronic flow in transistors and molecular flow in chemical reactions caused by their obeying the same laws of thermodynamics. (*b*) The figure shows that this similarity enables circuits in both domains to be mapped to each other.

There are striking similarities between chemical-reaction dynamics (figure 3*a*) and electronic current flow in the subthreshold regime of transistor operation (figure 3*b*): electron concentration at the source is analogous to reactant concentration; electron concentration at the drain is analogous to product concentration; forward and reverse current flows in the transistor are analogous to forward and reverse reaction rates in a chemical reaction; the forward and reverse currents in a transistor are exponential in voltage differences at its terminals analogous to reaction rates being exponential in the free-energy differences in a chemical reaction; increases in gate voltage lower energy barriers in a transistor increasing current flow analogous to the effects of enzymes or catalysts in chemical reactions that increase reaction rates; and the stochastics of the Poisson shot noise in subthreshold transistors are analogous to the stochastics of molecular shot noise in reactions. Some of these analogies have been listed in the table 3.

**Table 3.** Chemistry and electronics.

chemical-reaction dynamics	electron flow in transistor
reactant concentration	electron concentration at the source
product concentration	electron concentration at the drain
forward and reverse reaction rates in chemical reaction	forward and reverse current flows in the transistor
forward and reverse chemical reaction rates are exponential in the free-energy difference between transition state and reactant/product, respectively	forward and reverse electronic currents are exponential in the voltage difference between transistor channel and source/drain, respectively
enzymes or catalysts in chemical reactions increase reaction rates	increases in gate voltage lower energy barriers in a transistor, increasing current flow
stochastics of molecular Poisson processes in chemical reactions [1,11]	stochastics of electronic Poisson processes in subthreshold transistors [1,11]
flux balance analysis	Kirchoff's current law
chemical energy conservation	Kirchoff's voltage law
chemical concentration	current
electrochemical potential: log(concentration) + energy	electrochemical potential: log(current) + voltage

The logarithmic dependence of the electrochemical potential in chemical concentration or of current enables one to map log-domain analog transistor circuit motifs in electronics to log-domain analog molecular circuit motifs in cells and vice versa.

If we examine the classic Michaelis–Menten enzyme–substrate binding basis function, we find that
4.1
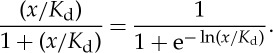
Therefore, if we look at the left-hand side, the basis function may be viewed as being approximately linear and proportional to (*x*/*K*_d_) for *x*<*K*_d_ and saturating after *x*=10 *K*_d_; or, if we look at the right-hand side, it may be viewed as being log-linear over the range 0.1*K*_d_<*x*<10*K*_d_ where the sigmoid is linear. Hence, this basis function has a log-linear analog regime of operation over the central portion of the sigmoid and a saturating digital regime at the extremes of the sigmoid. Note that ln(*K*_d_) is proportional to the free energy of the binding reaction. Highly compact eight-transistor log-domain analog differential-pair circuits generate almost exactly identical mathematical basis functions in either the linear current [21] (left-hand side of equation (4.1)) or log-linear voltage domain [1] (right-hand side of (4.1)). Log-domain analog circuits can generate any polynomially linear or polynomially nonlinear dynamical system in both electronics and in chemistry [1]. In particular, they can generate dynamical systems of the form
4.2
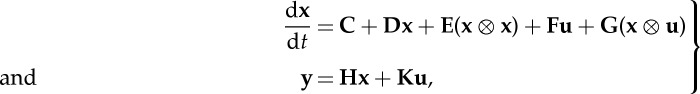
where **x** and **u** are vector state and input variables, respectively, the matrix coefficients are determined by chemical-reaction parameters, and the multiply operations refer to outer products derived from two-molecule chemical binding. The limitation to two-molecule binding is for practicality and leads to no loss of generality [1]: two molecules can bind to generate a complex, and the complex can then bind to the third as is true in most chemical reactions.

Figure 3*b* shows that the *cytomorphic* mapping between electronics and chemistry outlined in figure 3*a* and table 3 enables one to map from electronic circuits to DNA–protein circuits and vice versa. In this article, we focus on the mapping from electronic circuits to molecular circuits, which is most useful in synthetic biology. The other direction of mapping has been extensively discussed in [1] and is useful for the design and simulation of synthetic biological circuits or the ultrafast stochastic simulation of large-scale systems-biology circuits with supercomputing chips.

## 5. Logarithmic analog computation in living cells

To widen the dynamic range of input operation of an inducer, it is useful to have a wide log-linear range. Logarithmic transduction affords advantages such as constant-precision sensing at any intensity (Weber's law) and is seen in many natural systems, including audition, vision and in cells [49].

**Figure 4.**
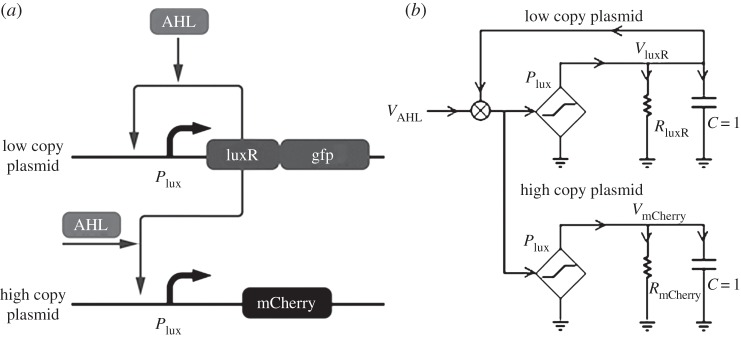
A positive-feedback linearization circuit. (*a*) A genetic circuit that linearizes inducer operation over a wide log-linear dynamic range is shown. (*b*) An analog circuit schematic represents this circuit.

If the concentration of a transcription factor is fixed, as the inducer increases in value, it will eventually be bound to all the available transcription-factor molecules and saturate the number of bound inducer–transcription-factor complexes that are possible. In addition, if the number of DNA-binding sites for a complex is limited, these sites will eventually all be bound by complexes and gene expression will saturate. These two sources of saturation limit the dynamic range of inducer operation. Figure 4*a* shows a genetic circuit motif [22] and an associated circuit schematic in figure 4*b* that simultaneously alleviates both these saturation problems to widen the dynamic range.

**Figure 5.**
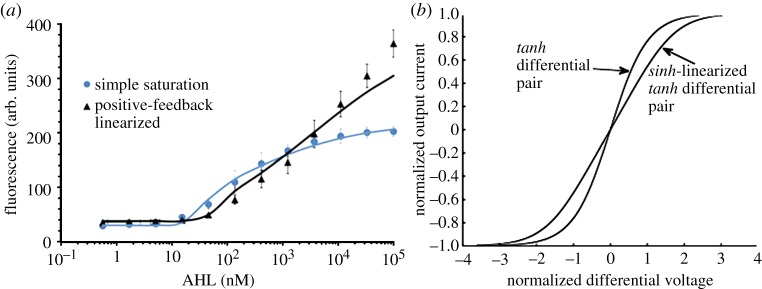
Linearization data. (*a*) The effect of linearization on the biological input is clearly seen. (*b*) A *sinh-linearized tanh* differential-pair circuit in electronics bears similarity to the genetic circuit motif. It also architects linearization by having expansive and compressive (saturating) nonlinearities interact. (Online version in colour.)

In figure 4*a*, a positive-feedback loop increases the generation of transcription factor by catalysing its own production on a low-copy plasmid. As the amount of inducer increases, more transcription factors are created alleviating the saturation of complexes. The high-copy plasmid has several binding sites that also shunt away the complexes, thus alleviating the saturation of DNA-binding sites on the low-copy plasmid. The high-copy plasmid also serves to control the gain of the positive-feedback loop by altering the number of complexes available to the low-copy plasmid [22]. Such control exploits ‘the fan-out loading’ of the downstream high-copy plasmid on the upstream low-copy plasmid as a desirable feature in our analog circuit. Fan-out is often a problem in molecular digital circuits. Figure 5*a* shows that the log-linear dynamic range is almost four orders of magnitude.

The genetic circuit of figure 4*a* actually exploits an idea from log-domain analog circuit design shown in figure 5*b*: expansive *sinh*-based linearization of compressive and saturating *tanh*-based differential-pair circuits causes them to widen their linear range of operation [24]. The function of the *sinh* is achieved by positive feedback in figure 4*a* and the function of the *tanh* is analogous to that of the saturation of biochemical binding. Indeed, in the future, other linearization circuit motifs from log-domain circuit design, e.g. those in [1], could also be exported to cells. The supplementary section of [22] models details how, analogous to the case in electronics [24], the widest dynamic range in the circuit motif of figure 4*a* is achieved at an optimal value of positive feedback.

**Figure 6.**
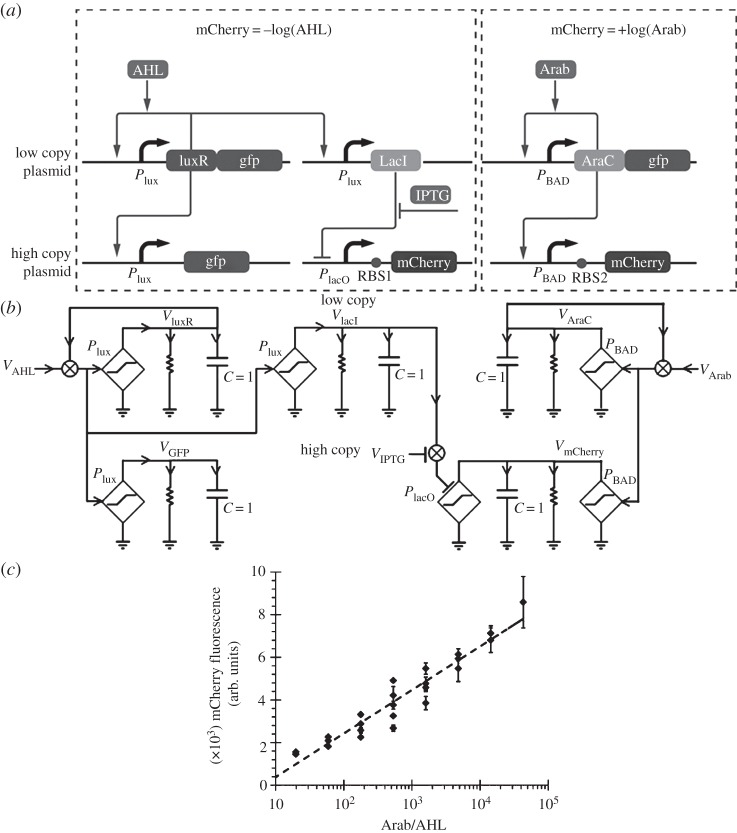
A pRATIO circuit. (*a*) The genetic circuit computes the logarithmic ratio of its two molecular inputs. (*b*) An associated electrical schematic equivalent is shown. (*c*) The ratio is computed over four orders of magnitude in the genetic circuit.

Figure 6*a* and its associated schematic in figure 6*b* show how to use logarithms to divide by using 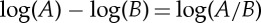: two linearized positive-feedback circuit motifs enable two inducers, Arab and AHL, to effectively control the expression of the mCherry fluorescent protein on the high-copy plasmid. The Arab input controls mCherry by *activating* its production. The AHL input generates LacI on the low-copy plasmid, which then *represses* mCherry production. The IPTG inducer fine-tunes this repression gain. The RBS1 and RBS2 translation gains (analogous to *g*_rib_ in figure 1*a*) also serve to fine-tune the gain of the positive logarithm w.r.t. the gain of the negative logarithm. The net result is that the logarithmic ratio of the two inducer molecules in figure 6*c* is obtained over almost four orders of magnitude. This ‘pRATIO’ circuit generalizes the concept of pH, a logarithmic ratio used to measure H^+^ concentration w.r.t. a reference, to any arbitrary ratio of two inputs w.r.t. one another. It may have applications for the wide-dynamic-range sensing of biomolecules by serving as a log differential amplifier.

In log-linear systems, the computational basis functions needed for universality besides the logarithm itself are addition, subtraction and scaling. Daniel *et al.* [22] shows how to architect logarithmic addition by merely summing up fluxes as in our analog adder. We have already shown how to subtract two logarithmic inputs in figure 6*a*,*b*. Scaling is also implicit in figure 6*a*,*b* (via the IPTG, RBS1 and RBS2 gains). The equivalent of addition, subtraction and scaling with logarithms corresponds to multiplication, division and power laws in the linear domain, much as in the operation of slide rules.

**Figure 7.**
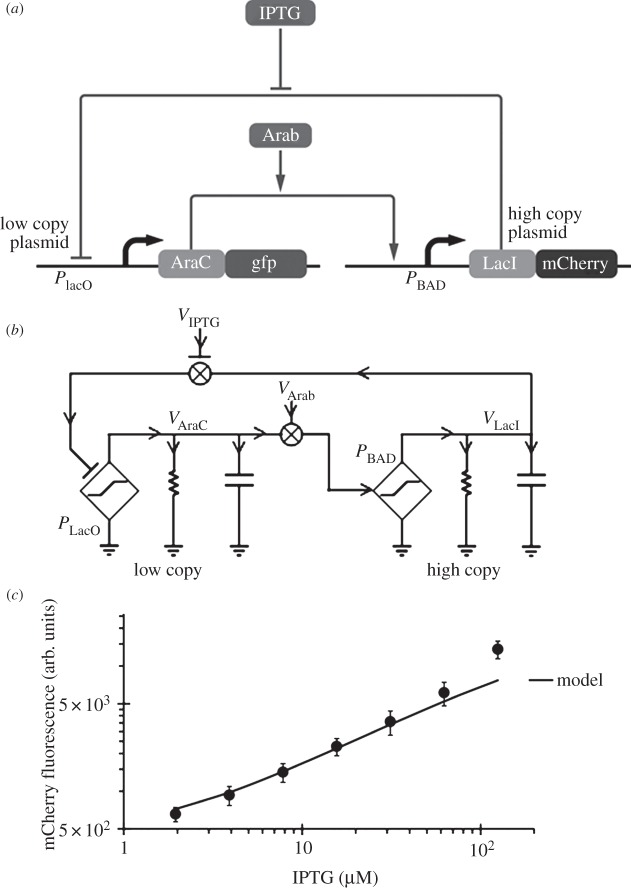
A power-law circuit. (*a*) A genetic power-law circuit. (*b*) An associated electrical equivalent schematic is shown. (*c*) The experimental data for the genetic circuit.

Figure 7*a* and its associated circuit schematic in figure 7*b* show how to compute a power law without any explicit use of logarithms: the high-copy plasmid produces a high level of LacI that strongly represses AraC production with AraC=*g*_AraC_(*K*_df_/LacI)(IPTG/*K*_*m*_)^*h*^; IPTG controls the strength of its de-repression according to the inherent power law *h*, and *g*_AraC_ is the gain of the first-stage amplifier in figure 7*b*. As the low-copy production of AraC does not saturate the binding sites on the high-copy plasmid, the falling AraC reduces the level of LacI in a relatively linear fashion according to LacI=*g*_LacI_ (AraC/*K*_d_), with *g*_LacI_ being the gain of the second-stage amplifier of figure 7*b*. The net consequence of the negative feedback is then that LacI must equilibrate at a consistent value, where
5.1
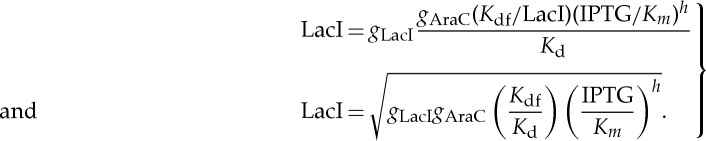
The net power law w.r.t. IPTG is then *h*/2, which in figure 7*c* is found to be about 0.7. The supplementary section in [22] provides further details. This power-law circuit only requires two transcription factors to implement, in contrast to an *in vitro* circuit, which required 130 DNA parts to implement a square-root circuit with 2 bits of output precision [23].

## 6. Summary

We summarize by reviewing seven benefits of analog computation in cells, which are important for synthetic (and systems) biology.

(1) *Digital computation is a subset of analog computation* that only operates at its saturated high or low extremes. Therefore, by allowing operation over the whole range of signal levels and by allowing exploitation of all the basis functions of biochemistry and biophysics, not just logic, *horizons for computation are expanded*. Digital computation will continue to be important for decision-making, signal restoration, communication and sequential operation.
(2) *At the moderate precision of computation seen in cells, analog computation is significantly more efficient in its use of energy, time and space* (molecular count and part count) than digital computation. Therefore, analog and collective analog computation is *likely to be more practical and scalable for synthetic biology* than purely digital computation.
(3) *Probabilistic digital computation operates with analog probabilities, and behaves as noisy analog computation*. As even digital computation in cells may be forced to be probabilistic to cope with constraints on part count, molecular count and energy, analog computation may be inevitable.
(4) *Problems such as loading and fan-out*, which are limiting the scaling of synthetic biology, *are part of the design process of analog computation*, which uses concepts of input and output impedance and feedback design to cope with such problems. It can even exploit them for computation (figure 4*a*,*b*).
(5) *Analog design*, which has been developed over several decades, *provides accurate pictorial motifs with abstraction* (figure 1*b*) unlike pure differential-equation design, but these abstractions *are not oversimplified* as in molecular logic design.
(6) The great similarities between neural and cellular computation suggest that *analog computation in the cell may have predated and pioneered analog computation in the brain*.
(7) The *deep cytomorphic connection between electronics and chemistry* via the common electrochemical potential *can enable log-domain analog electronic circuits to inspire new analog circuit motifs in cells* and vice versa. Therefore, future work in synthetic biology or in systems biology will be likely to benefit from the cytomorphic mapping (figure 3*a*,*b*).

## Appendix A. Costs of *N*-bit digital molecular addition

An *N*-bit digital adder uses one ‘half-adder’ logic circuit (addition of two binary numbers *A*_0_ and *B*_0_ with no carry) at its least significant bit (LSB) and *N*−1 ‘full-adder’ logic circuits (addition of two binary numbers *A*_*n*_, *B*_*n*_, and the carry *C*_*n*−1_ from the previous bit) at all other *N*−1 bits. This architecture outputs local binary answers at each bit stage and also propagates carries to neighbouring bit stages. The carries propagate such that the most significant bit (the ‘MSB’ stage at bit *N*−1 with LSB being at bit 0) is the very last stage to output its answer. This scheme of addition is really just a binary version of classic decimal addition. More complex schemes, for example Brent–Kung adders, can reduce carry propagation delays but are too complex in a molecular context and do not alter any of the conclusions of this paper. Therefore, we shall only consider simple addition of two positive numbers via the classic method of addition described above. We shall also not delve into two's complement arithmetic because we shall assume that all numbers are positive.

The half-adder stage implements the following logic for the local sum *S*_0_ and carry *C*_0_:

and

These logic functions can be implemented with two genetic promoter circuits that each implement a half-sided XOR (such circuits are common as a repressor and an activator often interact in genetic promoter circuits), one genetic OR promoter circuit, and one genetic AND promoter circuit. All of these two-input genetic circuits have been reported in the literature and are possible to synthesize [31]. We shall refer to the half-sided XOR circuit as an H-XOR. Three-input genetic circuits have thus far not been robust or easy to synthesize, so we shall not use them.

The full-adder stage at the *n*th bit implements the following logic for the local sum *S*_*n*_ and carry *C*_*n*_:

and

Thus, it requires 3×2 H-XORs that need to be composed in cascade (for *S*_*n*_), four ANDs (two cascaded for *S*_*n*_ and two for *C*_*n*_) and five ORs. In our scheme of architecting the digital-adder circuit, no signal inversions are necessary, which saves gates. However, it does assume a combinatorial set of promoters that respond to inverted and non-inverted logical signals as needed. Such promoters cost no power and add no molecular count in our accounting. Thus, we are likely to be underestimating digital resource consumption compared with an actual implementation.

Hence, if *N* is at least 2 (the 1-bit case is degenerate because there are no full-adder circuits) the net number of gates, each of whose molecular outputs leads to an energy-consuming synthesis operation, and the net number of molecular inputs, which also lead to energy-consuming synthesis operations, can be computed from table 4.

**Table 4.** Resource consumption in an *N*-bit digital adder.

LSB sum and	(*N*−1) MSB	*N* MSB	*N*-bit		
carry gates	sum gates	carry gates	inputs	gate	total
2	6(*N*−1)	0	0	H-XOR	6*N*−4
1	2(*N*−1)	2*N*	0	AND	4*N*−1
1	3(*N*−1)	2*N*	0	OR	5*N*−2
0	0	0	2*N*	input (no gate)	2*N*
				total molecules (no. of gate outputs plus inputs)	17*N*−7

As the final MSB output requires *N* stages of carry propagation, and the local sum and carry generation stages incur two logic-gate delays, the worst-case delay of the adder is approximately (*N*+2)*τ*_prot_, where *τ*_prot_ is protein degradation time constant of any logic gate.

For simplicity, we have assumed that there is no crosstalk between gates, which for a large number of gates may be difficult to architect in practice.
